# Exploring Major Flavonoid Phytochemicals from *Nelumbo nucifera* Gaertn. as Potential Skin Anti-Aging Agents: In Silico and In Vitro Evaluations

**DOI:** 10.3390/ijms242316571

**Published:** 2023-11-21

**Authors:** Bodee Nutho, Duangjai Tungmunnithum

**Affiliations:** 1 Department of Pharmacology, Faculty of Science, Mahidol University, Bangkok 10400, Thailand; bodee.nut@mahidol.ac.th; 2 Department of Pharmaceutical Botany, Faculty of Pharmacy, Mahidol University, Bangkok 10400, Thailand

**Keywords:** lotus plant, skin-aging enzyme inhibition, molecular docking, molecular dynamics simulation

## Abstract

*Nelumbo nucifera* Gaertn., an aquatic medicinal plant (Nelumbonaceae family), has a history of use in traditional medicine across various regions. Our previous study demonstrated the skin anti-aging potential of its stamen ethanolic extract by effectively inhibiting collagenase and tyrosinase enzymes. While the major constituents of this extract are well documented, there is a lack of research on the individual compounds’ abilities to inhibit skin aging enzymes. Therefore, this study aimed to evaluate the anti-aging potential of the primary flavonoids found in *N. nucifera* using both in silico and in vitro approaches. Our initial step involved molecular docking to identify compounds with the potential to inhibit collagenase, elastase, and tyrosinase. Among the seven flavonoids studied, kaempferol-3-*O*-robinobioside (Kae-3-Rob) emerged as the most promising candidate, exhibiting the highest docking scores for three skin aging-related enzymes. Subsequent enzyme-based inhibition assays confirmed that Kae-3-Rob displayed robust inhibitory activity against collagenase (58.24 ± 8.27%), elastase (26.29 ± 7.16%), and tyrosinase (69.84 ± 6.07%). Furthermore, we conducted extensive 200-ns molecular dynamics (MD) simulations, revealing the stability of the complexes formed between Kae-3-Rob and each enzyme along the MD simulation time. MM/PBSA-based binding free energy calculations indicated the considerably stronger binding affinity of Kae-3-Rob for collagenase and tyrosinase compared to elastase, which was related to the greater percentage of hydrogen bond occupations. These computational findings were consistent with the relatively high inhibitory activity of Kae-3-Rob against collagenase and tyrosinase observed in our in vitro experiment. In conclusion, the results obtained from this comprehensive study suggest that Kae-3-Rob, a key flavonoid from *N. nucifera*, holds significant potential as a source of bioactive compounds for anti-aging cosmeceutical and other phytopharmaceutical application.

## 1. Introduction

Skin aging is a multifaceted process characterized by various conditions, including the loss of skin elasticity and strength, along with the development of pigmentation disorders [1]. Furthermore, skin aging involves hyperpigmentation, the breakdown of collagen and elastin fibers, leading to the formation of wrinkles, skin laxity, dryness, and impaired wound healing [2]. These concerns have been associated with the increased activity of key aging-related enzymes such as collagenase and elastase [3,4]. Additionally, the level of melanin content and its distribution are considered important factors affecting skin color. Tyrosinase, the key rate-limiting enzyme, regulates melanin content during the process of melanogenesis. Unfortunately, abnormal melanin production causes various dermatological problems such as freckles, melasma, age spots, and senile lentigines, leading to flaws and a premature aging appearance. Therefore, inhibiting tyrosinase activity is a common approach recommended for addressing pigmentation disorders and is used as a whitening agent for aesthetic purposes [5]. While synthetic skincare products with anti-aging ingredients are available, they can sometimes lead to adverse reactions such as allergic contact dermatitis, irritant contact dermatitis, and phototoxic and photo-allergic responses [6]. Consequently, the research community is currently focusing on exploring phytochemicals extracted from medicinal plants and/or their major phytochemical compounds, especially flavonoids, as a promising avenue in addressing skin aging [7,8,9,10,11,12].

**Figure 1:**
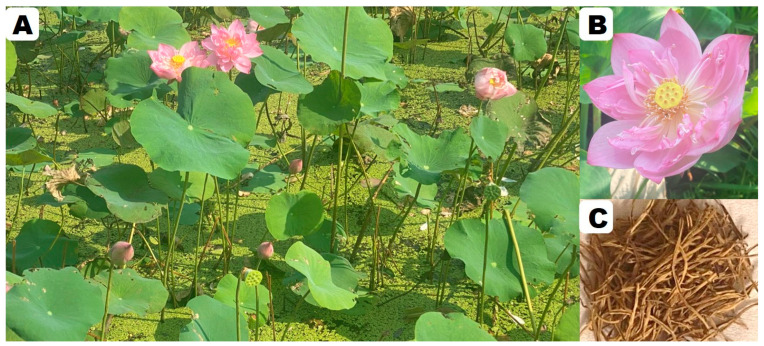
Sacred lotus: (**A**) natural habitat, (**B**) floral parts, (**C**) dried stamens, a part used for Thai traditional medicine and flavonoids rich part. The photo was taken in Thailand by Duangjai Tungmunnithum.

Sacred lotus is an Asian lotus species that is widely used for both ornamental and medicinal proposes [7,8,13,14,15,16,17]. Due to the beauty of its flowers, sacred lotus has long been used for ornamental purposes. This lotus is also recognized as the spiritual symbol of various religions such as Buddhism, Hinduism, and Ancient Egyptian religion since the ancient period [8,13,14,15]. This sacred lotus is a species member of an aquatic flowering plant family Nelumbonaceae (Figure 1), and its scientific name is also known as *Nelumbo nucifera* Gaertn. [13,14]. This species distributes mainly in Asian regions, e.g., Thailand, India, China, Sri Lanka, Nepal, and Japan [13,14,18,19,20]. Furthermore, sacred lotus is used as an ingredient for preparing various traditional medicines or herbal drugs [18,19,20,21,22,23].

Nowadays, a large number of research teams have studied phytochemical characterization as well as the pharmacological activities of this medicinal species [7,8,16,17,18,19,20,21,22,23,24,25,26,27]. The anti-aging effect of *N. nucifera*, in particular against degenerative diseases, has been recently ascribed to its flavonoid fraction [9,28,29,30]. In lotus species, the stamen is an enriched source of flavonoids [7,8,9,10,17,18,19,20,21,22,23,25,26,27,28,29,30]. The stamen ethanolic extract of *N. nucifera* contains seven major flavonoids, namely myricetin-3-*O*-glucoside (Myr-3-Glc), rutin, quercetin-3-*O*-glucuronide (Quer-3-Glu), kaempferol-3-*O*-robinobioside (Kae-3-Rob), kaempferol-3-*O*-glucoside (Kae-3-Glc), kaempferol-3-*O*-glucuronide (Kae-3-Glu), and isorhamnetin-3-*O*-glucoside (Iso-3-Glc) [7,8,9,19]. Our previous study demonstrated that the ethanolic extract from *N. nucifera* stamens by using ultrasound-assisted extraction methodology exhibited promising inhibitory effects on aging-related enzymes, specifically collagenase and tyrosinase, with a relatively weaker inhibition observed for elastase and hyaluronidase [9]. However, we have yet to explore the individual major compounds within this medicinal plant to assess their anti-aging activity against enzymes associated with skin aging.

This research aims to explore the anti-aging properties of major flavonoids found in *N. nucifera* stamen concerning three crucial enzymes associated with the skin aging process, such as collagenase, elastase, and tyrosinase, using computational approaches. In vitro enzyme-based assays were also performed to compare with these molecular modeling data. Herein, we initially screened the flavonoids against these three aging-related enzymes using the molecular docking technique. The most potent compound, as determined by its superior docking score among the three enzymes, underwent experimental testing through in vitro enzyme inhibition assays. Subsequently, molecular dynamic (MD) simulation was conducted to gain insights into the dynamic behavior of the protein–ligand complex in aqueous solution, which provides the mechanism of action at the atomic level. As far as we know, this study is the first report on the molecular modeling of the major flavonoids found in the *N. nucifera* medicinal plant. Ultimately, we anticipate that these findings will be the fundamental data for the further development of new anti-aging cosmeceuticals or phytopharmaceutical applications.

## 2. Results and Discussion

### 2.1. Molecular Docking

**Figure 2:**
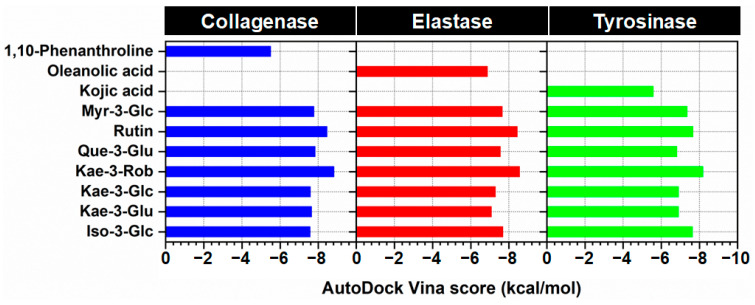
Docking results calculated with AutoDock Vina scoring function of seven major compounds from *N. nucifera* toward collagenase, elastase, and tyrosinase relative to their reference compounds (1,10-phenanthroline, oleanolic acid, and kojic acid).

To predict the potency of seven flavonoid glycosides against three aging-associated enzymes—collagenase, elastase, and tyrosinase—molecular docking was performed using AutoDock Vina 1.2.5. The docking scores of each compound with the target enzymes are presented in Figure 2, where lower values indicate better binding. Generally, all the major flavonoids from the *N. nucifera* stamen ethanolic extract displayed lower docking scores than positive controls for all target enzymes. Interestingly, Kae-3-Rob exhibited the highest binding affinity against the three enzymes (−8.82, −8.55, and −8.18 kcal/mol for collagenase, elastase, and tyrosinase, respectively) compared to the other compounds. The second most promising compound with anti-aging potential against these aging-related proteins was rutin. This finding is consistent with a previous report that highlighted the notable biological effects of rutin on skin aging. The anti-aging properties of rutin were determined using a cell viability assay, reverse transcription-quantitative polymerase chain reaction, senescence-associated-β-galactosidase assay, and reactive oxygen species scavenging activity in in vitro; the anti-aging of rutin in vivo model, rutin-containing cream, was tested in human skin with a double-blind clinical study [31].

**Figure 3:**
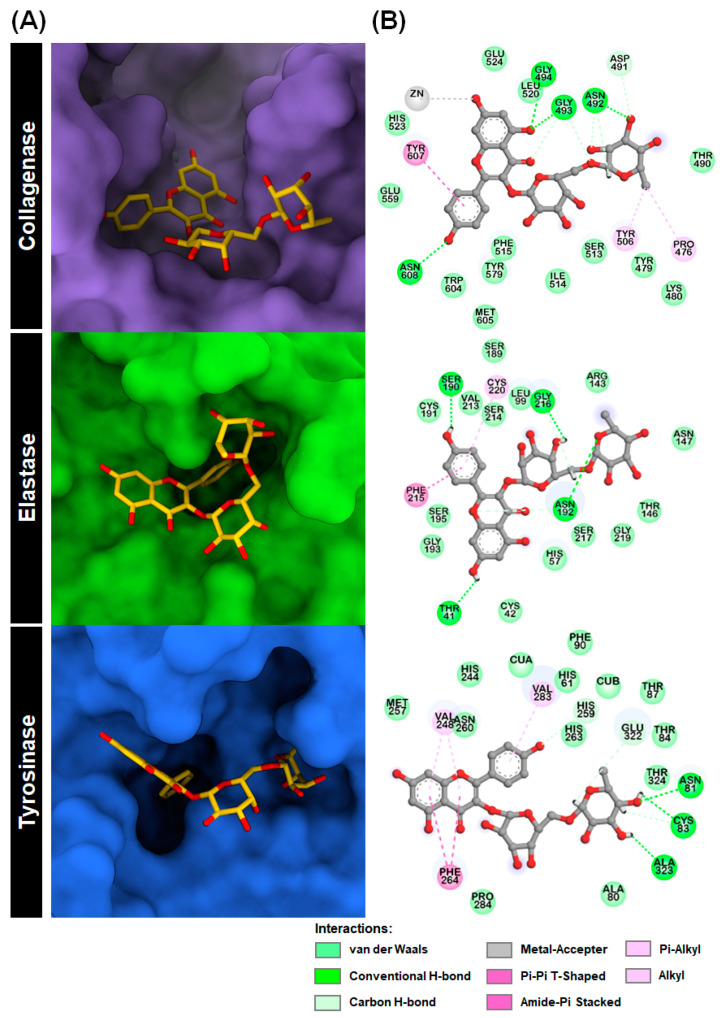
(**A**) Docked structures of Kae-3-Rob bound to collagenase, elastase, and tyrosinase along with (**B**) their 2D interaction diagrams.

From a docking perspective, Kae-3-Rob has gained much attention in this study. To the best of our knowledge, there are no reports on the inhibitory activity of this compound against these aging-related enzymes. To provide further insight, we analyzed the binding mode and interactions of Kae-3-Rob with each target enzyme obtained from molecular docking. The 3D graphics and 2D diagrams of protein–ligand interactions are depicted in Figure 3.

Figure 3A illustrates the 3D and 2D representations of the most favorable binding pose of Kae-3-Rob on the collagenase catalytic domain. The results revealed that Kae-3-Rob can effectively bind to the enzyme’s active site during the docking process. The most stable conformation of Kae-3-Rob displayed interactions with several residues in the enzyme binding pocket. These interactions involve van der Waals (vdW), hydrogen bonding (H-bond), and π-related interactions. Furthermore, the catalytic Zn^2+^ ion, which plays a crucial role in collagenase’s enzymatic process, could form a metal–acceptor interaction with the 7-hydroxyl group on the chromone ring of Kae-3-Rob. This interaction likely resulted in the blocking of Zn^2+^ during catalysis and/or disturbed the interactions with zinc metal-binding residues (i.e., H523, H527, and E555) [32]. These findings may explain the strong inhibitory effect of Kae-3-Rob against collagenase, as determined by enzymatic assays (as shown further in Section 2.2).

In the molecular docking analysis of Kae-3-Rob with elastase, it was observed that the B-ring of Kae-3-Rob effectively occupied the active site of elastase, which includes the catalytic triad (H57, D102, and S195) [33]. This particular configuration was primarily stabilized by the H-bond with S190, amide-π stacking with F215, and π-alkyl interactions with C220. Additionally, residues T41, N152, and G216 were found to form H-bonds with the A-ring, rhamnose, and galactose moieties, respectively. Notably, our results indicate that while Kae-3-Rob could interact with multiple residues within the binding pocket of elastase, it did not form any direct interaction with the catalytic triad, only exhibiting weak vdW interactions with S195. These observations may explain the relatively weak inhibitory activity of Kae-3-Rob against elastase, as shown further in Section 2.2. This was in contrast to epigallocatechin gallate (EGCG), which could directly bind to the catalytic triad and exhibited potent inhibition [34].

The docked complex between Kae-3-Rob and tyrosinase is illustrated in Figure 3A. Kae-3-Rob displayed great potential for inhibiting tyrosinase, as indicated by its superior binding affinity compared to the positive control, kojic acid (docking score −5.57 kcal/mol). The molecular docking results revealed that ring B of Kae-3-Rob, which has a structural resemblance to the tyrosine substrate, effectively occupied the active site of mushroom tyrosinase, suggesting its ability to compete with tyrosine binding [35]. In the 2D interaction map, the chromone ring was stabilized through π-related interactions with the residues V248 (π-alkyl) and F264 (π-π T-shaped), while V283 participated in a π-alkyl interaction with ring B of Kae-3-Rob. Additionally, the hydroxyl groups on the rhamnose moiety of Kae-3-Rob formed conventional H-bonds with the binding site residues N81, C83, and A323. Although the compound did not directly interact with the binuclear copper ions (CuA and CuB) at the catalytic center, the 4′-hydroxyl group on the B-ring formed a non-classical carbon H-bond with H259, one of the coordinating residues with CuB. Furthermore, the B-ring established hydrophobic contacts with H61 and H263, which are coordinated with CuA and CuB, respectively. Therefore, our docking results suggest that Kae-3-Rob can disrupt the redox cycle critical for the catalytic activity of tyrosinase by binding into the catalytic cavity and interfering with the interactions between the active site copper ions and the histidine-based catalytic residues. This similar binding pattern within the tyrosinase catalytic domain, as derived from molecular docking, has also been observed for other phytochemicals, such as galangin [36], (+)-catechin [37], and luteolin 5-*O*-*β*-d-glucopyranoside [38].

### 2.2. In Vitro Assay of Kae-3-Rob toward Aging-Related Enzymes Inhibition

The results from the in vitro aging-related enzymes inhibition assay show that Kae-3-Rob, which is a major flavonoid phytochemical compound from the medicinal plant *N. nucifera* [7,8,9,19], exhibits anti-aging potential against the skin-aging enzymes (Table 1) including tyrosinase (69.84 ± 6.07% of enzyme inhibition), collagenase (58.24 ± 8.27% of enzyme inhibition), and elastase (26.29 ± 7.16% of enzyme inhibition), respectively. This result from this current study is consistent with what was previously reported on the potential of flavonoids as anti-aging phytochemical compounds for cosmetic, cosmeceutical, or phytopharmaceutical applications [7,31,39,40].

### 2.3. Molecular Dynamics of Kae-3-Rob Bound to Aging-Related Enzymes 

#### 2.3.1. System Stability

**Figure 4:**
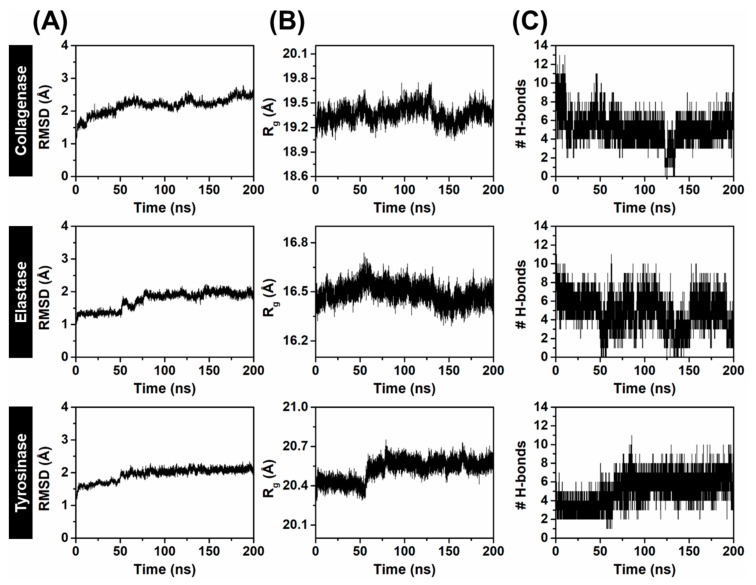
Time evolution of (**A**) RMSD, (**B**) R_g_, and (**C**) # H-bonds for Kae-3-Rob bound to collagenase, elastase, and tyrosinase during 200 ns MD simulations.

Since Kae-3-Rob showed the most negative binding energy against three aging-related enzymes (Figure 2), we further conducted 200 ns MD simulations to investigate the binding stability and dynamic behavior in an aqueous environment. To assess the stability of the simulated systems, the root-mean-square deviation (RMSD) of the complex atoms, including enzyme backbone atoms and ligand heavy atoms, was initially calculated over the course of the MD simulations. The RMSD profiles revealed that all the systems were found to reach an equilibrium after 125 ns (Figure 4A), with stable RMSD values ranging from 1.8 to 2.3 Å. Additionally, the compactness of the enzyme structure upon ligand binding was determined by calculating the radius of gyration (Rg) throughout the MD simulation time of 0–200 ns (Figure 4B). The Rg plots for protein C_α_ atoms showed that the three systems were relatively stable, with consistent Rg values of approximately 19.2–19.5 Å for collagenase, 16.4–16.6 Å for elastase, and 20.3–20.6 Å for tyrosinase. This indicated that the overall protein structures maintained their compactness throughout the MD simulation time. It is worth noting that a slight increase in the Rg value was observed after ~60 ns for the Kae-3-Rob–tyrosinase system, which could be attributed to minor adjustments in the enzyme’s structure when the ligand bound to its active site.

We also tracked the number of hydrogen bonds (# H-bonds) formed within each enzyme binding site and with Kae-3-Rob along the simulation times, as depicted in Figure 4C. A higher number of # H-bonds signifies the greater stability of the complex. In the Kae-3-Rob–collagenase complex, the time course of # H-bonds revealed consistent interactions from 50 to 200 ns, averaging around 6 H-bonds. Similarly, the H-bond profile of Kae-3-Rob bound to tyrosinase gradually increased from 0 to 80 ns, after which it remained constant (~5 H-bonds) until 200 ns. In contrast to both systems, the H-bond profile of the Kae-3-Rob–elastase system appeared to fluctuate (1–7 H-bonds) throughout the simulation, especially during the 50 to 150 ns period, suggesting that Kae-3-Rob may bind to the active site of elastase with less stability compared to collagenase and tyrosinase. Altogether, the structural parameters collectively indicate that throughout the MD simulations, all complexes remained stable, and there were no significant observed conformational changes. 

#### 2.3.2. Binding Affinity

Due to the consistency and small fluctuations observed in RMSD, Rg, and # H-bonds (Figure 4) during the 150–200 ns, the 500 MD snapshots extracted from this period were used to calculate the binding free energies ($∆G_{\mathrm{bind}}$) of the simulated systems. The binding affinities of Kae-3-Rob to collagenase, elastase, and tyrosinase were estimated using the Molecular Mechanics-Poisson Boltzmann surface area (MM/PBSA) approach. The method is considered as more computationally accurate compared to docking scoring functions (empirical or knowledge-based scoring functions), as it can detect conformational changes induced by ligand binding and provide a rigorous free energy decomposition, offering insights into contributions from various atom groups and types of interactions [41]. The $∆G_{\mathrm{bind}}$, together with its energy components of each system, is given in Table 2. Note that the normal mode analysis [42] that was conducted to calculate the entropic contribution (T∆S) averaged over only 50 snapshots, due to the substantial computational cost associated with the calculation.

The calculated MM/PBSA energies for Kae-3-Rob binding to collagenase, elastase, and tyrosinase were as follows: −6.30, −2.71, and −8.93 kcal/mol, respectively. More specifically, the corresponding energetic values revealed that the electrostatic term ($∆E_{\mathrm{ele}}$) was the primary contributor to the binding energies of the Kae-3-Rob–collagenase complex, surpassing the vdW term ($∆E_{\mathrm{vdW}}$) by ~1.5-fold. This phenomenon could be attributed to the relatively high # H-bonds formed between the protein and ligand, as depicted in Figure 4. In contrast, the Kae-3-Rob–elastase complex was primarily stabilized by the $∆E_{\mathrm{vdW}}$ term, while the $∆E_{\mathrm{ele}}$ and $∆E_{\mathrm{vdW}}$ values were closely similar for the tyrosinase system. However, when considering solvation free energies, the polar term ($∆E_{\mathrm{ele}}+∆G_{\mathrm{sol}}^{\mathrm{ele}}$) resulted in an unfavorable binding contribution (positive value), as opposed to the favorable non-polar term ($∆E_{\mathrm{vdW}}+∆G_{\mathrm{sol}}^{\mathrm{nonpolar}}$). This pattern is commonly observed in the binding of various protein–ligand complexes [43,44,45]. Note that all three complexes exhibited nearly identical values for the T∆S contribution (~−22 kcal/mol). In summary, these findings indicated a stronger binding affinity of Kae-3-Rob to collagenase and tyrosinase compared to elastase. This may justify the in vitro anti-aging assays, where Kae-3-Rob showed more potent inhibition against collagenase and tyrosinase than against elastase.

#### 2.3.3. Key Binding Residues

**Figure 5:**
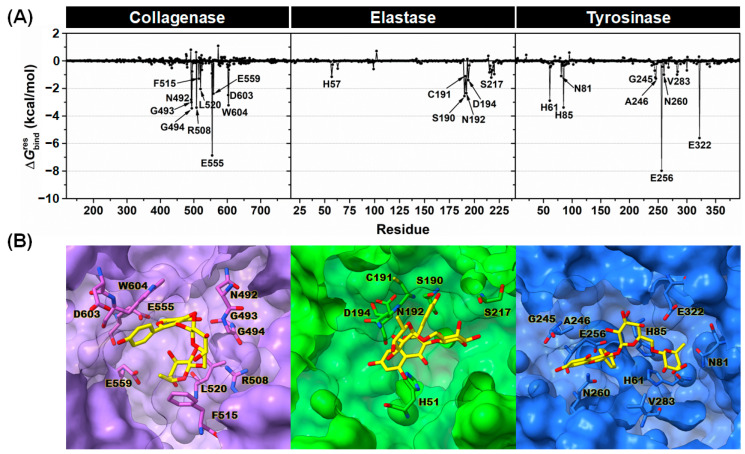
(**A**) The plots of $∆G_{\mathrm{bind}}^{\mathrm{res}}$ calculated with the MM/PBSA method for Kae-3-Rob complexed with collagenase, elastase, and tyrosinase, with the key residues involved in ligand binding labeled in the graph (energy stabilization of ≤−1.0 kcal/mol). (**B**) A close-up view of key influential residues of collagenase, elastase, and tyrosinase contributing to Kae-3-Rob binding.

To further analyze the crucial binding residues of collagenase, elastase, and tyrosinase important for Kae-3-Rob binding, the calculation of MM/PBSA per residue decomposition energy ($∆G_{\mathrm{bind}}^{\mathrm{res}}$) for each complex system was conducted. This analysis was performed over the same set of 500 snapshots used for the binding free energy calculations mentioned above. The results obtained are presented in Figure 5A, where the residues demonstrating an energy stabilization of ≤−1.0 kcal/mol are marked in the plots. The binding orientations of Kae-3-Rob within the active site of each enzyme are depicted in Figure 5B.

**Figure 6:**
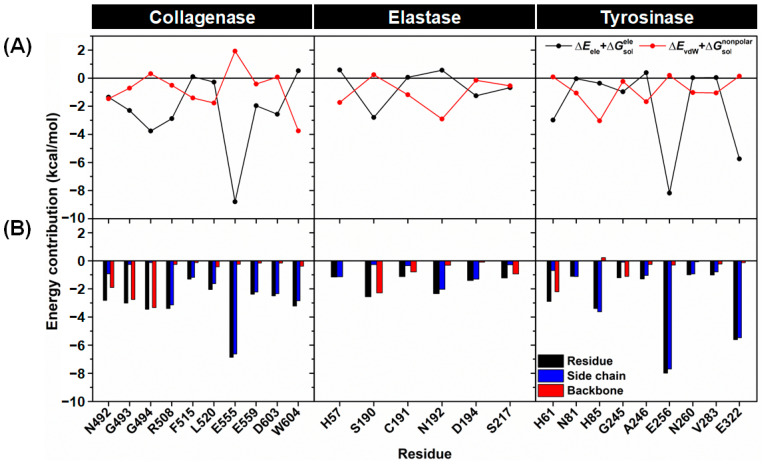
(**A**) Energy contribution from polar ($∆E_{\mathrm{ele}}+∆G_{\mathrm{sol}}^{\mathrm{ele}}$, black line) and nonpolar ($∆E_{\mathrm{vdW}}+∆G_{\mathrm{sol}}^{\mathrm{nonpolar}}$, red line) terms from each residue of collagenase, elastase, and tyrosinase to the binding of Kae-3-Rob. (**B**) $∆G_{\mathrm{bind}}^{\mathrm{res}}$ was expressed as total (black bars), side chain (blue bars), and backbone (red bars) contributions for the binding of Kae-3-Rob to collagenase, elastase, and tyrosinase.

The critical residues in collagenase that participated in binding with Kae-3-Rob included N492, G493, G494, R508, F515, L520, E555, E559, D603, and W604. These stabilizing amino acid residues were also implicated in the binding of other reported collagenase inhibitors such as ohioensin A, *nor*-ohioensin D [46], bisresorcinol [47], and turmerone [48]. Notably, residue E555, which is one of the Zn^2+^-binding residues, exhibited the most negative energy contribution ($∆G_{\mathrm{bind}}^{\mathrm{res}}$ of −6.9 kcal/mol) to ligand binding, primarily through electrostatic interactions (as discussed later in Figure 6). This interaction likely disrupts the binding with the catalytic Zn^2+^ during catalysis, thereby inhibiting the enzymatic activity. Consistent with this observation, residue E555 has also been identified as forming a strong H-bond with EGCG, a potent collagenase inhibitor [49]. In the case of the Kae-3-Rob–elastase complex, six stabilizing residues (H57, S190, C191, N192, D194, and S217) were associated with the binding of Kae-3-Rob to elastase. While Kae-3-Rob could directly bind to the catalytic residue H57 through hydrophobic interactions (as shown in Figure 6), the relatively low energy contribution (−1.1 kcal/mol) may not be sufficient to outcompete substrate binding and enzyme catalysis. This observation aligns with the weaker inhibition of Kae-3-Rob against elastase (Table 1). In contrast, EGCG was capable of direct interactions with the catalytic residues of elastase, leading to strong enzyme inhibition [34]. Meanwhile, the binding of Kae-3-Rob with tyrosinase revealed the key binding residues crucial for ligand binding, including H61, N81, H85, G245, A246, E256, N260, V283, and E322. Specifically, Kae-3-Rob interacted with the catalytic histidine residues H61 and H85, potentially interfering with the active CuA catalytic center. Furthermore, E256 and E322, with favorable energy contributions (−7.9 and −5.6 kcal/mol, respectively), played an essential role in maintaining the position of the B-ring and rhamnose moiety within the tyrosinase binding pocket. The significance of both residues in the ligand binding process has also been observed in other reported mushroom tyrosinase inhibitors, such as bromophenols [50], caffeine [49], and carvacrol derivatives [51].

**Figure 7:**
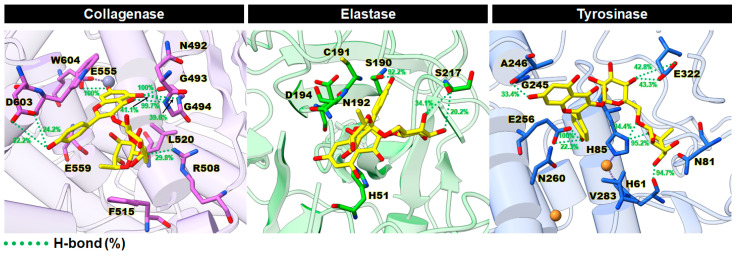
Representative 3D structures showing the percentage of H-bond occupations (green dotted lines) of Kae-3-Rob with the active site residues of collagenase, elastase, and tyrosinase.

The degree of stabilization from the individual residues highlighted in Figure 5 was further considered in terms of the polar ($∆E_{\mathrm{ele}}+∆G_{\mathrm{sol}}^{\mathrm{ele}}$) and nonpolar ($∆E_{\mathrm{vdW}}+∆G_{\mathrm{sol}}^{\mathrm{nonpolar}}$) energies, as shown in Figure 6A. The contributed energies from the backbone and side chain atoms of each residue were also analyzed (Figure 6B). The results showed that the electrostatic interactions from the collagenase residues G493, G494, R508, E555, E559, and D603 predominantly contributed to the binding of Kae-3-Rob. This finding aligns with the molecular mechanics energy ($∆E_{\mathrm{MM}}$, Table 2), as well as the H-bond formations detected in this complex (Figure 7, discussed later). In contrast, the primary energy contribution for the binding between elastase and Kae-3-Rob mainly came from the nonpolar energy for H57, C191, and N192. However, for S190, the importance of this residue in recognizing the Kae-3-Rob binding was through polar interactions (i.e., H-bonding, Figure 7). In the Kae-3-Rob–tyrosinase system, it was found that the energy contributions of each residue primarily came from nonpolar interactions (N81, H85, A246, N260, and V283), while polar interactions were preferred for H61, E256, and E322. It is worth noting that most of the key binding residues of each enzyme likely stabilized Kae-3-Rob through their side chains, as evidenced by the greater energy contribution (more negative values) from the side chain (blue bars) than from the backbone (red bars), as shown in Figure 6B. On the other hand, the backbone atoms of the collagenase (N492, G493, and G494), elastase (S190, C191, and S217), and tyrosinase (H61 and G245) residues played a crucial role in stabilizing the binding with Kae-3-Rob.

#### 2.3.4. Intermolecular Hydrogen Bonds (H-Bonds)

It is well established that H-bonds play a pivotal role in protein folding and protein–ligand interactions, as they tightly anchor the molecule in the enzyme active site [52]. Thus, the percentage of H-bond occupations between Kae-3-Rob and each enzyme was measured over the last 50 ns using the geometric criteria outlined in the materials and methods (see Section 3.1.3). The representative 3D structures with the corresponding percentage of H-bond occupations for each system are illustrated in Figure 7. In the Kae-3-Rob–collagenase complex, H-bond formations indicated a strong stabilization (>80% occupation) with the residues G493 (100%), G494 (99.7%), and E555 (100%). This aligns with the preferential electrostatic interactions mentioned earlier (Figure 6A). These H-bonds likely contribute to maintaining the chromone ring of Kae-3-Rob inside the collagenase active site. In contrast, the Kae-3-Rob–elastase complex exhibited only one strong H-bond with the residue S190 at 92.2%, which corresponds to the weaker $∆E_{\mathrm{ele}}$ term compared to the $∆E_{\mathrm{vdW}}$ energy (Table 2). In the case of the Kae-3-Rob–tyrosinase complex, three strong H-bonds were detected with H61 (94.7%), H85 (95.2%), and E256 (100%). These interactions helped stabilize the B-ring and sugar moiety within the binding pocket of tyrosinase. Taken altogether, our findings suggest that H-bond formations played a key role in the binding of Kae-3-Rob with collagenase and tyrosinase, in contrast to elastase.

## 3. Materials and Methods

### 3.1. Computational Studies

#### 3.1.1. System Preparation and Molecular Docking

The three-dimensional (3D) structure of collagenase G from *clostridium histolyticum* (PDB ID: 2Y6I [32]), pancreatic porcine elastase (PDB ID: 1BRU [53]), and tyrosinase from *Agaricus bisporus* (PDB ID: 2Y9X [54]) were retrieved from the RCSB Protein Data Bank. The solvent molecules and co-crystallized ligands were removed from all selected protein structures. The missing residues (No. 598–600) in the collagenase structure were constructed using the SWISS-MODEL server [55]. The protonation states of all ionizable amino acids of the target protein were predicted at pH 7.4 using the H++ web server [56]. Meanwhile, the SMILES format of Myr-3-Glc (CID: 44259426), rutin (CID: 5280805), Quer-3-Glu (CID: 5274585), Kae-3-Rob (CID: 15944778), Kae-3-Glc (CID: 5282102), Kae-3-Glu (CID: 5318759), and Iso-3-Glc (CID: 5318645) were taken from the PubChem database (https://pubchem.ncbi.nlm.nih.gov, accessed on 9 August 2023), as provided in Table S1 (Supplementary Materials). Next, the SMILES strings of each compound were converted into 3D PDB format utilizing the Online SMILES Translator and Structure File Generator (https://cactus.nci.nih.gov/translate, accessed on 9 August 2023). All the ligands were fully optimized at the B3LYP/6-31G(d) level using Gaussian09 program (Gaussian, Inc., Wallingford, CT, USA) [57]. Finally, the prepared protein structures and optimized ligands were changed into the PDBQT file format using the AutoDockFR 1.0 software suite [58] before performing molecular docking.

Molecular docking studies were executed by AutoDock Vina 1.2.5 [59]. The crystalized ligand of each protein structure was defined as the docking site. The dimensions of the grid box size were equally set to 24 Å for collagenase, 20 Å for elastase, and 26 Å for tyrosinase. The grid center x, y, and z coordinates had the following values: (i) 24.1, −2.7, and 15.9 (collagenase), (ii) 23.2, 47.7, and 17.1 (elastase), and (iii) −10.0, −28.8, and −43.6 (tyrosinase). The exhaustiveness value was set to 64, while the remaining parameters were kept at the program’s default values. All screened compounds were ranked by binding energy (in kcal/mol) based on the AutoDock Vina scoring function (a more negative value indicates higher affinity). Among the three proteins, the docked complex with the lowest AutoDock Vina docking score (i.e., the best pose) was chosen as the initial configuration for MD simulation. Furthermore, the 3D binding mode of the protein–ligand complex was visualized using the UCSF Chimera [60] and ChimeraX [61] programs, while the 2D diagram of protein–ligand interactions was verified using the Discovery Studio Visualizer (BIOVIA, San Diego, CA, USA).

#### 3.1.2. Molecular Dynamic (MD) Simulations

The MD simulations of the protein–ligand complexes were run under the periodic boundary condition with the isothermal–isobaric (NPT) ensemble using SANDER and PMEMD modules of the AMBER 20 software package (University of California, San Francisco, CA, USA) [62]. Prior to performing MD simulations, ligand parameters in terms of the partial atomic charges and empirical force field were generated as follows. The electrostatic potential (ESP) charges of the optimized geometry (see above) were computed by single-point calculation at the HF/6-31G(d) level of theory. Further, the antechamber module of AMBER20 was employed to convert the ESP charges of the ligand to the restrained ESP (RESP) charges. The missing molecular parameters of the ligand were derived from the general AMBER force field 2 (GAFF2) [63] using the parmchk2 module. The AMBER ff14SB force field [64] was applied to the protein. All missing hydrogen atoms of the protein–ligand complex were added by the LEaP module. The catalytic Zn^2+^ and Cu^2+^ ions of the respective collagenase and tyrosinase were treated using the 12-6-4 Lennard-Jones-type non-bonded model developed by Li and Merz [65]. Each system was immersed in a simulation box of the TIP3P explicit solvation model [66] with a minimum buffer thickness of 10 Å. The sodium (Na^+^) or chloride (Cl^−^) counterions were randomly added to neutralize the total charges of the systems. To relax the structure, the added hydrogen atoms and solvent molecules were minimized using the steepest descent (SD) and conjugated gradient (CG) methods of 3000 and 1000 iterations, respectively. Finally, the entire system was energetically minimized with 3000 steps of SD and 1000 steps of CG methods. A 10-Å cutoff distance and the particle mesh Ewald (PME) method [67] were used to treat the non-bonded interactions and long-range electrostatic interactions, respectively. An integration time step of 2 fs was applied for MD simulation in combination with the SHAKE algorithm [68] to constrain all covalent bonds involving hydrogen atoms. To maintain the target temperature and pressure of the simulated systems, the Langevin thermostat [69] with a damping frequency of 2 ps^−1^ and the Berendsen barostat [70] with a pressure-relaxation time of 1 ps were conducted. 

Each simulated system was gradually heated from 10 to 300 K over 200 ps using a canonical ensemble (NVT) with positional restraints of 30.0 kcal/mol·Å^2^ to the binding residues within a 5-Å sphere from the ligand. Afterward, the complex was subjected to NPT equilibration of restrained MD simulations with a slowly decreased force constant of 30, 20, 10, and 5 kcal/mol·Å^2^ for 10 ns in total, and another 1000 ps without any restraint. Subsequently, the pre-equilibrated systems were simulated under the NPT scheme at 300 K and 1 atm until reaching 200 ns. The MD trajectories were collected every 10 ps.

#### 3.1.3. Post-Dynamic Trajectories Analyses

To investigate the structural variations of the simulated systems, the structural parameters, including RMSD, Rg, and the #H-bonds between protein and ligand, were calculated. Note that the #H-bonds and H-bond occupation analysis were monitored using the following geometric criteria: (i) the distance between the hydrogen donor (HD) and acceptor (HA) ≤ 3.5 Å and (ii) a HD–H···HA angle of ≥120°. In addition, $∆G_{\mathrm{bind}}$ calculations based on the MM/PBSA method [71] were utilized to predict the binding affinity of the protein–ligand complexes. Meanwhile, $∆G_{\mathrm{bind}}^{\mathrm{res}}$ was calculated to verify the amino acid residues of each protein crucial for ligand binding. Both $∆G_{\mathrm{bind}}$ and $∆G_{\mathrm{bind}}^{\mathrm{res}}$ were performed on 500 snapshots extracted from the last 50 ns of each MD simulation. The structural information and binding free energies were computed using the CPPTRAJ utility [72] and MMPBSA.py module [73] of AMBER 20, respectively.

### 3.2. In Vitro Anti-Aging Activity

#### 3.2.1. Chemicals

All solvents employed in this study were of analytical grade, provided by Thermo Scientific (Waltham, MA, USA). Standards and reagents were obtained from Sigma-Aldrich (St. Louis, MO, USA).

#### 3.2.2. Collagenase Assay

The collagenase *clostridium histolyticum* (Sigma-Aldrich) was used for this study, and its activity was determined with the aid of a spectrophotometer (Shimadzu, Kyoto, Japan) using N-[3-(2-furyl)acry loyl]-Leu-Gly-Pro-Ala (FALGPA; Sigma-Aldrich) as the substrate in accordance with the protocol of Wittenauer et al. [74]. The decrease in the absorbance of the FALGPA was followed for 20 min at 335 nm using a microplate reader (BMG labtech, Mornington, VIC, Australia). The measurements were conducted in triplicate, and the anti-collagenase activity was revealed as the percent inhibition relative to the control for every sample. The specific inhibitor of collagenase used was 1,10-Phenantroline (100 μM).

#### 3.2.3. Elastase Assay

The elastase assay was performed using porcine pancreatic elastase (Sigma-Aldrich), and its activity was investigated with a spectrophotometer (Shimadzu, Japan) using N-Succ-Ala-Ala-Alap-nitroanilide (AAAVPN; Sigma-Aldrich) as the substrate and following p-nitroaniline’s release at 410 nm using a microplate reader (BMG labtech, Australia) modified based on the method described by Wittenauer et al. [74]. The measurements were conducted in triplicate, and the anti-elastase activity was revealed as the percent of inhibition relative to the control for every sample. Oleanolic acid (10 μM) was used as the specific inhibitor of elastase.

#### 3.2.4. Tyrosinase Assay

The tyrosinase assay was conducted following the method described by Chai et al. [75]. Briefly, L-DOPA (5 mM; Sigma-Aldrich) was used as the diphenolase substrate, and then mixed in sodium phosphate buffer (50 mM, pH 6.8) with 10 μL of the sample. Lastly, 0.2 mg/mL of mushroom tyrosinase solution (Sigma-Aldrich) was added to this mixture in order to reach the final volume of 200 μL. The reaction processes were detected using a microplate reader (BMG labtech, Australia) at a wavelength of 475 nm. Tyrosinase’s inhibitory effect was expressed as the percent of inhibition relative to the control. Kojic acid (10 μM) was used as the specific inhibitor of tyrosinase.

## 4. Conclusions

In this study, we investigated the anti-aging potential of seven major flavonoid glycosides from the ethanolic extract of *N. nucifera* stamen against three critical skin aging-related enzymes: collagenase, elastase, and tyrosinase, employing a combination of computational and experimental approaches. The docking results obviously identified Kae-3-Rob as the compound with the highest docking scores for all three enzymes. Subsequent in vitro enzyme-based assays confirmed Kae-3-Rob’s notable inhibition of collagenase and tyrosinase, albeit with weaker inhibition against elastase. To gain a comprehensive understanding of the structural dynamics and molecular interactions involved in Kae-3-Rob’s binding to each enzyme, we conducted extensive 200-ns MD simulations. These simulations revealed the stability of each system throughout the duration of the simulations, as supported by calculations of RMSD, Rg, and # H-bonds. Furthermore, binding free energy calculations, utilizing the MM/PBSA method, consistently indicated a notably stronger binding affinity of Kae-3-Rob when complexed with collagenase and tyrosinase compared to elastase, which was in good agreement with the experimental results. Moreover, our analysis highlighted the significant role of H-bond formations in facilitating the binding of Kae-3-Rob with collagenase and tyrosinase. Overall, our study provides the first evidence that Kae-3-Rob, which is a major flavonoid from *N. nucifera* stamen, can potentially act as a promising collagenase and tyrosinase inhibitor. For the direction of further research, these current findings illustrate the potential of Kae-3-Rob as an alternative choice for future anti-aging cosmetic and cosmeceutical product development, but the safety of the product needs to be confirmed. For its application in phytopharmaceutical products or herbal drugs, the human-derived information, in vivo models, as well as clinical trials should be evaluated in future research.

## Figures and Tables

**Table 1:** Comparison of in vitro skin aging enzyme inhibition of Kae-3-Rob.

Anti-Aging Activity	% of Enzyme Inhibition ^a^
Tyrosinase	69.84 ± 6.07
Collagenase	58.24 ± 8.27
Elastase	26.29 ± 7.16

^a^ 1,10-Phenantroline (100 µM) was used as the specific inhibitor of collagenase, leading to an inhibition of 33.4 ± 1.9%, while kojic acid (10 µM) was used as the specific inhibitor of tyrosinase, leading to an inhibition of 51.2 ± 0.9%. Oleanolic acid (10 µM) was used as the specific inhibitor of elastase, leading to an inhibition of 46.7 ± 1.5%.

**Table 2:** The average $∆G_{\mathrm{bind}}$ and its energy component (kcal/mol) of Kae-3-Rob bound to collagenase, elastase, and tyrosinase calculated with the MM/PBSA method. Data are shown as means ± the standard error of the mean (SEM).

	Collagenase	Elastase	Tyrosinase
$∆E_{\mathrm{vdW}}$	−28.96 ± 0.27	−35.38 ± 0.14	−32.02 ± 0.18
$∆E_{\mathrm{ele}}$	−46.08 ± 0.25	−13.23 ± 0.17	−35.34 ± 0.36
$∆E_{\mathrm{MM}}$	−75.04 ± 0.27	−48.61 ± 0.21	−67.36 ± 0.29
−T∆S	22.57 ± 2.02	21.71 ± 1.03	21.72 ± 1.84
$∆G_{\mathrm{sol}}^{\mathrm{ele}}$	50.61 ± 0.22	28.25 ± 0.18	41.36 ± 0.24
$∆G_{\mathrm{sol}}^{\mathrm{nonpolar}}$	−4.44 ± 0.01	−4.06 ± 0.01	−4.64 ± 0.01
$∆G_{\mathrm{sol}}$	46.17 ± 0.21	24.19 ± 0.17	36.71 ± 0.24
$∆G_{\mathrm{total}}$	−28.87 ± 0.20	−24.42 ± 0.12	−30.65 ± 0.14
$∆G_{\mathrm{bind}}$	−6.30	−2.71	−8.93

## Data Availability

Data are contained within the article and supplementary materials.

## Supplementary Materials

The supporting information can be downloaded at: https://www.mdpi.com/article/10.3390/ijms242316571/s1.
