# Supplementary material for: Exploring Major Flavonoid Phytochemicals from Nelumbo nucifera Gaertn. as Potential Skin Anti-Aging Agents: In Silico and In Vitro Evaluations
Source: Int J Mol Sci. 2023 Nov 21;24(23):16571. doi: 10.3390/ijms242316571 (PMC10706394; doi:10.3390/ijms242316571)
Supplement: Supplementary file 1 [file ijms-24-16571-s001.zip › ijms-2716576-supplementary.pdf]

Supporting Material

Exploring Major Flavonoid Phytochemicals from *Nelumbo nucifera* Gaertn. as Potential Skin Anti-aging Agents: *In Silico* and *In Vitro* Evaluations

Bodee Nutho [1] and Duangjai Tungmunnithum [2,*]

[1] Department of Pharmacology, Faculty of Science, Mahidol University, Bangkok 10400, Thailand; bodee.nut@mahidol.ac.th
[2] Department of Pharmaceutical Botany, Faculty of Pharmacy, Mahidol University, Bangkok 10400, Thailand
* Correspondence: duangjai.tun@mahidol.ac.th

Table S1. Molecular structure of seven flavonoid glycosides in smiles format.

Compound	Smiles format
Myr-3-Glc	C1=C(C=C(C(=C1O)O)O)C2=C(C(=O)C3=C(C=C(C=C3O2)O)O)O[C@H]4C(C([C@@H]([C@H](O4)CO)O)O)O
Rutin	C[C@H]1[C@@H]([C@H]([C@H]([C@@H](O1)OC[C@@H]2[C@H]([C@@H]([C@H]([C@@H](O2)OC3=C(OC4=CC(=CC(=C4C3=O)O)O)C5=CC(=C(C=C5)O)O)O)O)O)O)O)O
Quer-3-Glu	C1=CC(=C(C=C1C2=C(C(=O)C3=C(C=C(C=C3O2)O)O)O[C@H]4[C@@H]([C@H]([C@@H]([C@H](O4)C(=O)O)O)O)O)O)O
Kae-3-Rob	C[C@H]1[C@@H]([C@H]([C@H]([C@@H](O1)OC[C@@H]2[C@@H]([C@@H]([C@H]([C@@H](O2)OC3=C(OC4=CC(=CC(=C4C3=O)O)O)C5=CC=C(C=C5)O)O)O)O)O)O)O
Kae-3-Glc	C1=CC(=CC=C1C2=C(C(=O)C3=C(C=C(C=C3O2)O)O)O[C@H]4[C@@H]([C@H]([C@@H]([C@H](O4)CO)O)O)O)O
Kae-3-Glu	C1=CC(=CC=C1C2=C(C(=O)C3=C(C=C(C=C3O2)O)O)O[C@H]4[C@@H]([C@H]([C@@H]([C@H](O4)C(=O)O)O)O)O)O
Iso-3-Glc	COC1=C(C=CC(=C1)C2=C(C(=O)C3=C(C=C(C=C3O2)O)O)O[C@H]4[C@@H]([C@H]([C@@H]([C@H](O4)CO)O)O)O)O
